# Transgenerational influence of parental morphine exposure on pain perception, anxiety-like behavior and passive avoidance memory among male and female offspring of Wistar rats

**DOI:** 10.17179/excli2019-1845

**Published:** 2019-11-05

**Authors:** Hamid Ahmadian-Moghadam, Mitra-Sadat Sadat-Shirazi, Fereshteh Seifi, Saba Niknamfar, Ardeshir Akbarabadi, Heidar Toolee, Mohammad-Reza Zarrindast

**Affiliations:** 1 Iranian National Center for Addiction Studies, Tehran University of Medical Sciences, Tehran, Iran; 2 Biology Department, Faculty of Biological Sciences, Islamic Azad University, North Tehran Branch, Tehran, Iran; 3 Department of Veterinary Medicine, Garmsar Branch, Islamic Azad University, Garmsar, Iran; 4 Department of Anatomy, School of Medicine, Tehran University of Medical Sciences, Tehran, Iran; 5 Department of Pharmacology, School of Medicine, Tehran University of Medical Sciences, Tehran, Iran; 6 Endocrinology and Metabolism Research Institute, Tehran University of Medical Science, Tehran, Iran

**Keywords:** drug administration, epigenetic, behavior, morphine

## Abstract

Accumulating evidence suggests that epigenetic mechanisms play an important role in the formation and maintenance of memory within the brain. Moreover, the effect of parental drug-exposure before gestation on behavioral state of offspring has been little studied. The main objective of the current study is to evaluate the effect of parental morphine exposure on avoidance memory, morphine preference and anxiety-like behavior of offspring. The total of 32 males and 32 females were used for mating. The animals were treated with morphine. The offspring according to their parental morphine treatment was divided into four groups (n=16) including paternally treated, maternally treated, both of parents treated and naïve animals. The pain perception, anxiety-like behavior, and avoidance memory were evaluated in the offspring. In the current study, the total of 256 offspring was used for the experiments (4 tasks × 4 groups of offspring × 8 female offspring × 8 male offspring). The finding revealed that the avoidance memory and visceral pain were reduced significantly in male and female offspring with at least one morphine-treated parent. Moreover, anxiety-like behavior was reduced significantly in the male offspring with at least one morphine-treated parent. While anxiety-like behavior was increased significantly in female offspring that were treated by morphine either maternally or both of parents. The data revealed that the endogenous opioid system may be altered in the offspring of morphine-treated parent(s), and epigenetic role could be important. However, analysis of variance signified the important role of maternal inheritance.

## Introduction

Drug addiction has a strong genetic component and it has a link to environmental variables (Tsuang et al., 1998[70]; Karkowski et al., 2000[40]; Kendler et al., 2000[41]; Nielsen et al., 2012[52]; Kenny 2014[42]). Moreover, the addiction has predicted the heritability in the range of 30 to 70 percent (Yohn et al., 2015[76]; Goldberg and Gould, 2019[30]). It was suggested that there is a link between drug exposure and drug-induced behavior in the exposed individuals (Agrawal and Lynskey 2008[2]; Tuesta and Zhang, 2014[71]). Morphine is the most efficient drug for suppression of the musculoskeletal and surgical pain (Johannes et al., 2010[37]). Moreover, the rewarding effect of morphine is related to the dopamine neural activity (Zarrindast et al., 2002[78]; Heidari et al., 2006[33]; Byrnes et al., 2013[16]). The effect of drug is mediated by opioid receptors. Moreover, Dopamine neurons activated by morphine release through disinhibiting the GABA neurons located in the VTA and modulating both hippocampal serotonin and cortical noradrenaline, that is known to be involved in learned helplessness paradigm and pathogenesis of anxiety (Yoshioka et al., 1993[77]; Besson et al., 1996[12]; Sastre-Coll et al., 2002[61]). 

The epigenetic mechanisms such as DNA methylation, histone modification are involved in drug addiction (Robison and Nestler 2011[58]; Kanherkar et al., 2014[38]). The chronic opioid misuse through epigenetic transmission may impair germ cell development in the addicted phenotypes (Chorbov et al., 2011[18]; Chidambaran et al., 2017[17]). The accumulative evidence suggests that the behavioral changes of addicted individuals is under control of epigenetics factors. For instance, the evidence showed that the nociception could be related to the intensity of the pain stimulus which is under control of epigenetic and genetic factors (Elmer et al., 1998[24]; Ashabi et al., 2018[7]). Another evidence showed that epigenetic mechanisms may play an important role in memory maintenance, memory storage and consolidation (Bruijnzeel et al., 2004[15]; Day and Sweatt 2011[23]; Wang et al., 2012[73]; Crist et al., 2013[21]). However, the role of epigenetic factors on memory formation is not clear (Tuesta and Zhang, 2014[71]). In addition, morphine has an interactive effect on memory formation in the passive avoidance memory test (Zarrindast and Rezayof, 2004[79]). Moreover, anxiety is comorbid with addiction, and chronic drug use can exacerbate the severity of the anxiety and increase the amount of drug taking (Bruijnzeel et al., 2004[15]; Crist et al., 2013[21]). In other words, drugs of abuse are able to usurp pain memory systems through their direct pharmacological actions on multiple neurotransmitter systems (Schultz, 2010[63]). 

Moreover, the effect of drugs of abuse on sexes is different based on the way their bodies absorb, distribute and metabolize the substance (Sanchis-Segura and Becker, 2016[59]). Sex-differences as a biological factor interact with epigenetic, genetics and environmental factors to mediate pathways for expression of special traits and it is considered important for addiction studies (Becker et al., 2017[11]). Furthermore, in neuroscience researches, female rats are not included due to the hormonal excretion and reproductive cycle. Although, it has been reported that in the animal model, females are not more variable than males. However, in the field of toxicology, this concern is still existing that the female rats due to estrous cycle generate tremendous variability (Fields, 2014[26]; Becker and Koob, 2016[10]). Moreover, scientific basis for medical decisions is based on the data collected from the male animals, and the achievement of the personalized medicine for females has been reduced. Furthermore, behavioral changes based on sex following morphine exposure rarely has been discussed. Regarding this background, there is a lack of knowledge on the transgenerational effect of parental morphine exposure on the behavioral state of the offspring. Moreover, this study appears to be a novel study evaluating the effect of morphine exposure before mating on avoidance memory, nociception, and anxiety-like behavior in both sexes in the offspring. 

## Material and Methods

### Animals

All experimental procedures were in agreement with the regulations of the experimental animal ethics at Tehran University of Medical Sciences ethics committee. Wistar albino rats, weighing between 200 to 220 grams were purchased from Pasture Institute, Tehran, Iran. The rats were exposed to morphine according to a modified protocol of Akbarabadi and colleagues (2018[3]). The total of 32 males and 32 females were used for mating and morphine treatment. The total of 128 male and 128 female offspring was used for the experiments (4 tasks × 4 groups of offspring × 8 female × 8 male). The animals were maintained in Plexiglas cages (n=4) with free access to fresh water and food at constant temperature 22±2 ºC and light/dark cycle (07:00-19:00 h). The biological father was removed before the birth of the offspring and the biological mother was kept until the end of the breastfeeding period. The experiments were started when offspring was reached to 8 weeks old. Moreover, female rats were tested on the diestrous phase of the estrous cycle and vaginal smear test was monitored daily. Moreover, to avoid potential confounding factory the offspring were no over-represented in the experiments. Furthermore one week before starting of the experiment the animals were picked up daily to reduce handling anxiety (Gouveia and Hurst, 2013[31]). 

### Drugs

In this study morphine sulfate (Temad Co., Tehran, Iran), naloxone hydrochloride (Sigma-Aldrich), and sucrose (Merck) were used. 

### Parental morphine exposure

**Figure 1:**
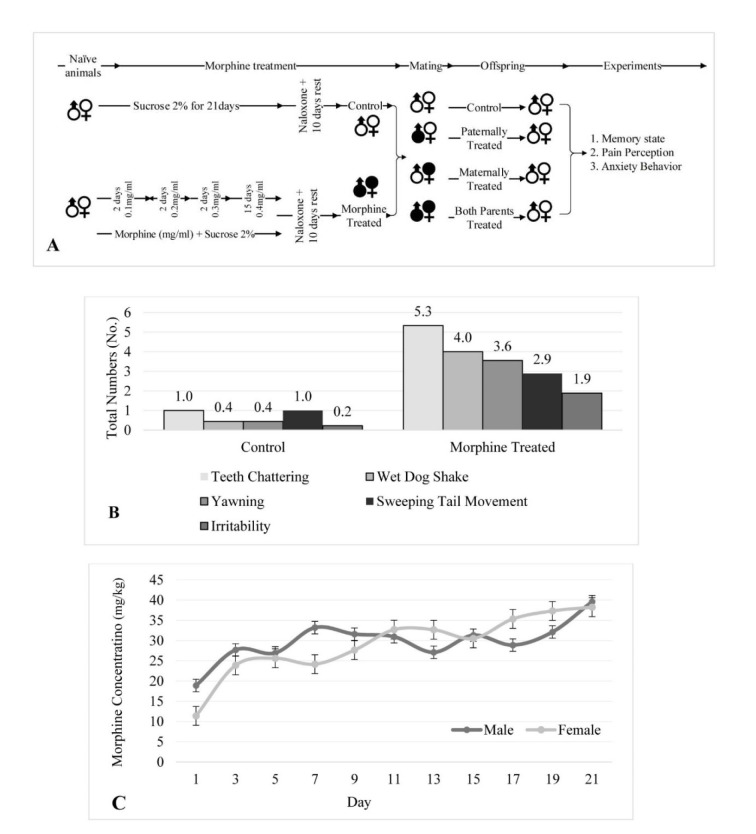
(A) The schematic diagram shows the study protocol for morphine exposure, mating, and final experiments. The offspring was divided to distinct groups as offspring of the control group (Control), offspring that maternally were treated by morphine (Maternally Treated), offspring that paternally were treated by morphine (Paternally Treated) and offspring that both parents were treated by morphine (Both Parents Treated). (B) The diagram shows withdrawal symptoms after naloxone in control and abstinent animals which confirm animals are in the morphine-abstinent state (P < 0.01). (C) The diagram shows milligram of morphine consumption per kilogram of rats within 21 days of morphine treatment.

Twenty-four male and twenty-four female Wistar rats were exposed to the treatment of oral morphine sulfate according to the protocol described earlier (Akbarabadi et al., 2018[3]). Morphine was given in the drinking fluid in the range of 0.1 to 0.4 mg.ml^-1^ in 48 intervals for up to three weeks (Figure 1A(Fig. 1)). Sucrose (2 %) was added to diminish the bitter taste of morphine. Eight male and eight female rats were considered as the control group which only received sucrose (2 %). Naloxone was administrated intraperitoneally (IP) for all of the animals to confirm the morphine dependence. Withdrawal symptoms and the average of morphine consumption were recorded (Figure 1B and 1C(Fig. 1)). 

### Mating protocol

Ten days after the last morphine administration, the animals were assigned for mating. The offspring of the animals was arranged in four groups as offspring of healthy parents (naive), offspring of morphine-treated female and healthy male rats (maternally treated), offspring of morphine-treated male and healthy female rats (paternally treated) and offspring of morphine-treated male and female rats (both of parents were treated).

### Avoidance memory 

#### Passive-avoidance memory test

The passive avoidance memory is evaluated according to the protocol that was described earlier (Akbarabadi et al., 2018[3]). The avoidance memory apparatus consists of a box with two compartments that were separated by a guillotine door. In the learning trial, the animal was placed in the light compartment and was allowed to cross the dark compartment, the guillotine door was closed and received an electric shock via gird floor (50 Hz, 1 mA, and 5s). The latency time of each animal to cross to the dark compartment was recorded. The experiment was repeated and in case no entrance to the dark compartment within 120s, a successful acquisition of avoidance memory was recorded. The animals with successful acquisition were subjected to the test trial. In the test trial, each animal was placed in the light compartment and the door was opened. The step-through latency for crossing to the dark compartment was recorded for each animal. The testing trial ended either when the animal entered into the dark compartment or remained in the light compartment until the cut-off time (300 s).

### Anxiety-like behavior 

#### Open-field test

The open-field test is a behavioral test to evaluate anxiety-like behavior based on the locomotor activity (Damián et al., 2014[22]; Motaghinejad et al., 2016[47]). The open-field apparatus consists of a plexiglass square box with walls to reduce outside noise and light. Each rat was placed at the center of apparatus and left to move freely for 10 min. The number of times that the animal preened its fur or tail with its mouth or forepaws (grooming), square crossed (locomotion) and the number of times that a rat reared up on its hind limbs (rearing) were recorded. After each monitor, the cage was cleaned with 70 % ethanol solution and left to dry. 

#### Forced swimming test

The rats that were used for the open-field test were re-used in the forced swimming test. Forced swimming test is conducted to assess anxiety-like behavior acording to the protocol described earlier (Porsolt et al., 1977[55]). Briefly, the rat was placed on a Plexiglas cylinder (60 × 30 cm) that was filled with water (25 °C) to the height of 30 cm. In trial day the rats were habituated to the environment by swimming in the cylinder for 5 min in 24 h before test day. In the test day each rat was allowed to swim for 5 min while a video camera recorded from above. The latency to immobility and the total time of immobility were measured as an index of anxiety-like behavior.

### Pain perception 

#### Writhing test

The writhing test is a method to evaluate nociception. Each animal was placed on a small observation chamber. After 10 min habituation a volume of 10 ml.kg^-1^ acetic acid (0.8 %) was i.p administrated. The nociceptive behavior characterized by abdominal contraction known as writhing, which is described as an exaggerated extension into the abdomen combined with the outstretching of hind limbs. Five minutes following the administration, the number of writhing and total time of writhing was recorded over 10 minutes (Singh et al., 1983[65]).

#### Formalin test

The formalin test was performed to evaluate acute and chronic pain. The pain is induced by applying 0.1 ml of 2.5 % formalin (Merck, Germany) into the dorsal surface of the left hind paw of each rat, and the rats were placed in an observation chamber with a mirror mounted on three sides to allow a clear view of the paws. The total times that each rat spent licking the injected paws were recorded. Acute pain as a result of nociceptor stimulation was observed in 1-10 min interval and the persistent pain was observed in 20-40 min interval of formalin injection (Hunskaar et al., 1985[35]). 

### Statistical analysis 

The chance of false positive results was reduced by normality analysis using Kolmogorov-Smirnov (K-S) test. The normal data with K-S value more than 0.01, were subjected to two-way ANOVA analysis followed by Dunnett's post-hoc mean comparison test. The p-values lower than 0.05 was considered significant. All statistical analyses were conducted using IBM SPSS 21 software. The results of the statistical analysis were summarized in Table 1(Tab. 1). 

## Results

### Avoidance memory

**Figure 2:**
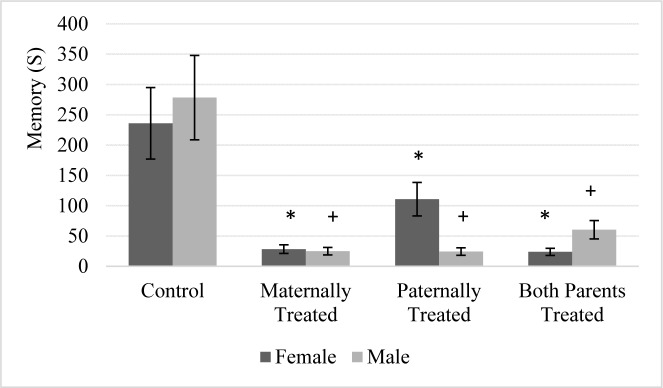
The figure shows the mean of each group for the avoidance memory of rats within passive avoidance memory test. Results of ANOVA test revealed there is no significant difference between sexes in the avoidance memory test. The average of each group was represented in offspring of the control group (Control), offspring that maternally were treated by morphine (Maternally Treated), offspring that paternally treated by morphine (Paternally Treated) and offspring that both parents were treated by morphine (Both Parents Treated). Results of post-hoc analysis revealed that in comparison with the control group, the avoidance memory in the female offspring with (*) sign significantly was reduced (p< 0.05). Moreover, results of post-hoc analysis revealed that in comparison with the control group, the avoidance memory in the male offspring with (+) sign significantly was reduced (p< 0.05).

Results showed that there is no significant difference between sexes for the avoidance memory. However, in comparison to control group the avoidance memory in the offspring with at least one morphine exposed parent significantly was reduced (F (3, 56) = 57.55, P < 0.01). The results of mean comparison showed that the avoidance memory in the offspring with at least morphine exposed parent significantly was reduced. While, there was no significant difference between groups of offspring that at least one of their parents was exposed to morphine (Figure 2(Fig. 2)). 

Furthermore, the interaction between sexes and offspring was significant which indicates female and male rat within groups of offspring were significantly different for avoidance memory (F (3, 56) = 4.41, P < 0.01). Moreover, the lowest avoidance memory was observed in male offspring that either paternally or maternally were treated by morphine, while lowest avoidance memory was observed in female offspring that either parents or paternally were treated by morphine (Figure 2(Fig. 2)).

### Anxiety-like behavior

#### Open-field test

Results showed there is a significant difference in the locomotion between sexes (F (1, 56) = 84.30, P < 0.05) and offspring (F (3, 56) = 6.89, P < 0.05). Results of mean comparison showed that in average the locomotion in the offspring of which both parents or maternally were exposed to morphine significantly was increased. 

**Figure 3:**
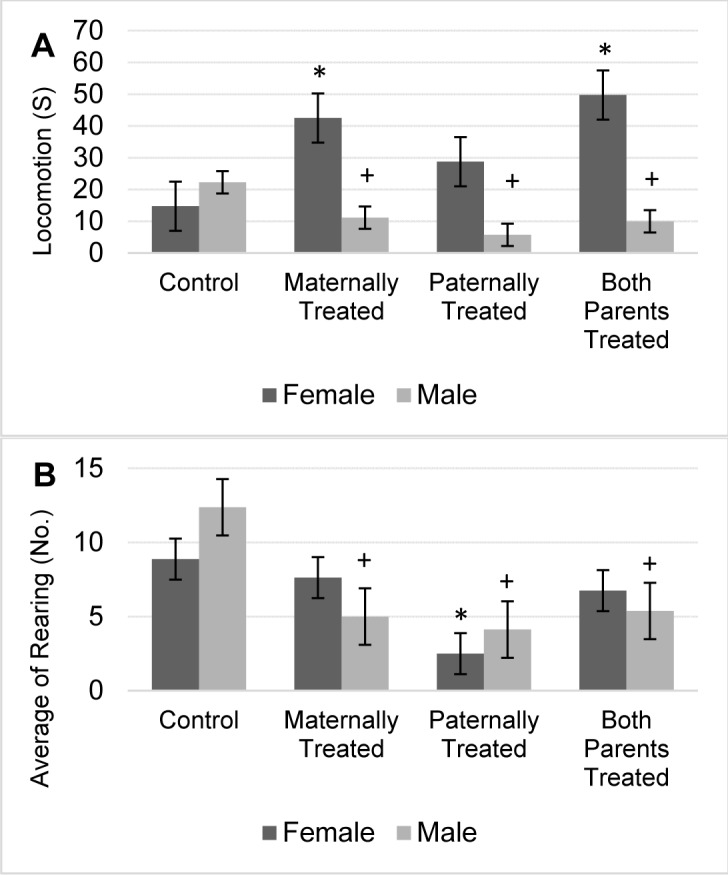
The figure shows the mean of each group for the locomotion (A) and rearing (B) of rats within the open-field test. Results of ANOVA test revealed there is a significant difference between sexes for locomotion of rats in the open-field test (F (1, 56) = 84.3, p< 0.05). However, the analysis revealed that there is no significant difference between sexes in the rearing behavior of rats in the open-field test. The average of each group was represented in offspring of the control group (Control), offspring that maternally were treated by morphine (Maternally Treated), offspring that paternally were treated by morphine (Paternally Treated) and offspring that both parents were treated by morphine (Both Parents Treated). Results of post-hoc analysis revealed that in comparison with the control group, the average of rearing and locomotion in the male offspring with (+) sign significantly were reduced (p< 0.05). Moreover, results of post-hoc analysis revealed that in comparison with the control group, the average of rearing and locomotion in the female offspring with (*) sign significantly was reduced (p< 0.05).

However, the interaction between sexes and offspring was significant which indicates female and male rat react significantly different within groups of offspring (F (3, 56) = 19.08, P <0.01). Results showed that the locomotion in male offspring with at least one morphine-treated parent significantly was reduced. However, the locomotion in female offspring that paternally or maternally were exposed to morphine significantly increased (Figure 3A(Fig. 3)). Moreover, no significant difference was observed in the locomotion of female offspring that paternally was exposed to morphine. However, in this group, the lowest locomotion was observed in male offspring (Figure 3A(Fig. 3)). Moreover, the results revealed that highest motor move was observed in female offspring that paternally or maternally were treated by morphine, that may suggest the role of maternal inheritance for change in locomotion (Figure 3A(Fig. 3)). 

The result showed that there is no significant difference in the rearing behavior between sexes. However, there is a significant difference in the total number of rearing between groups (F (3, 56) = 8.59, P < 0.05). Results showed that in male offspring with at least one morphine-treated parent, the average of the total number of rearing significantly reduced. However, in female offspring that paternally were treated by morphine a significant reduction in the average number of rearing was observed (Figure 3B(Fig. 3)). Furthermore, results showed that the lowest number of rearing was observed in male and female offspring paternally were treated by morphine (Figure 3B(Fig. 3)). 

#### Forced swimming test

**Figure 4:**
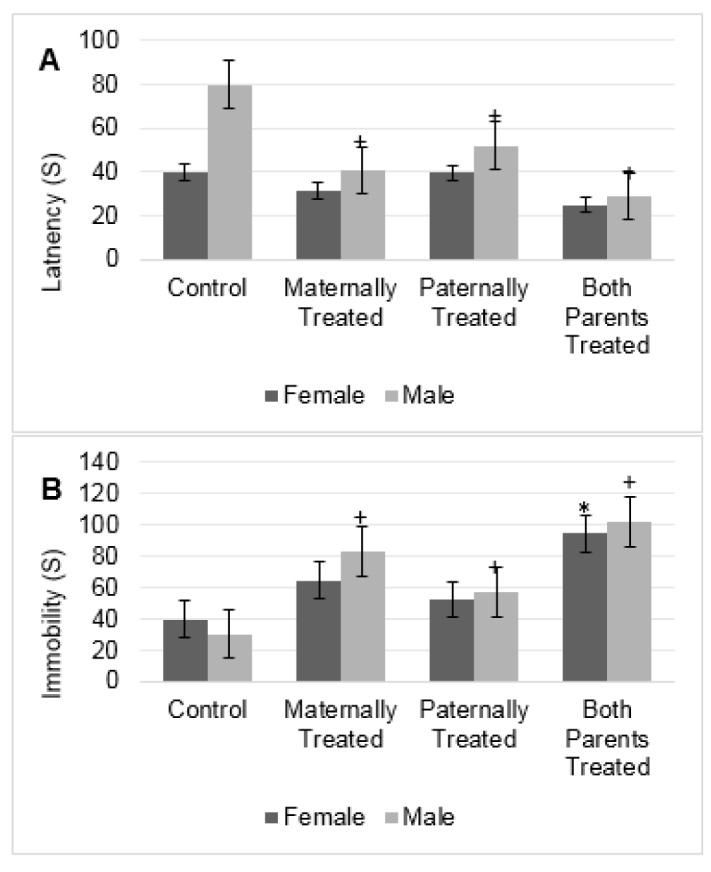
The figure shows the mean of each group for the latency (A) and immobility (B) of rats within the forced swimming test. Results of ANOVA test revealed that there is a significant difference between sexes for the latency of the rats in the forced swimming test (F (1, 56) =32.0, p< 0.05). However, the analysis revealed that there is no significant difference between sexes for immobility of rats in the forced swimming test. The average of each group was represented in offspring of the control group (Control), offspring that maternally were treated by morphine (Maternally Treated), offspring that paternally were treated by morphine (Paternally Treated) and offspring that both parents were treated by morphine (Both Parents Treated). Results of post-hoc analysis revealed that in comparison with the control group, the average of immobility and latency in the male offspring with (+) sign significantly changed (p< 0.05). Moreover, results of post-hoc analysis revealed that in comparison with the control group, the average of immobility in the female offspring with (*) sign significantly increased (p< 0.05).

The results showed that for the latency to immobility there is a significant difference between sexes (F (1, 56) = 32.00, P < 0.01) and offspring (F (3, 56) = 23.15, P < 0.01). The results of mean comparison test showed that in average the latency to immobility in the offspring with at least one morphine exposed parent significantly was reduced and the latency to immobility in the groups of offspring that both-parents or maternally exposed to morphine was lower in comparison to other groups (Figure 4A(Fig. 4)). 

Furthermore, the interaction between sexes and offspring was significant (F (3, 56) = 7.51, P <0.01). In male offspring with at least one morphine-treated parent, the latency to immobility significantly was reduced, while in female offspring no significant difference was observed (Figure 4A(Fig. 4)). Moreover, the highest latency to immobility was observed in male offspring that paternally were treated by morphine while the lowest once was observed in male offspring that both parents were treated by morphine (Figure 4A(Fig. 4)).

The results showed that there is no significant difference between the sexes for the total time immobility. 

However, the results showed there is a significant difference among the groups for the total time of immobility (F (3, 56) = 27.0, P < 0.05). The results of mean comparison showed that in average the total time of immobility in the offspring with at least one morphine exposed parent significantly was increased. However, in comparison to other groups the total time of immobility in the offspring that both parents or maternally exposed to morphine was higher (Figure 4B(Fig. 4)). 

Results revealed that in comparison with the control group, male offspring with at least one morphine-treated parent had a higher time of immobility (Figure 4B(Fig. 4)). Female offspring that both parents were treated by morphine, higher time of immobility was observed. The highest total time of immobility also was observed in male and female offspring that both parents were treated by morphine (Figure 4B(Fig. 4)).

### Pain perception

#### Writhing test

**Figure 5:**
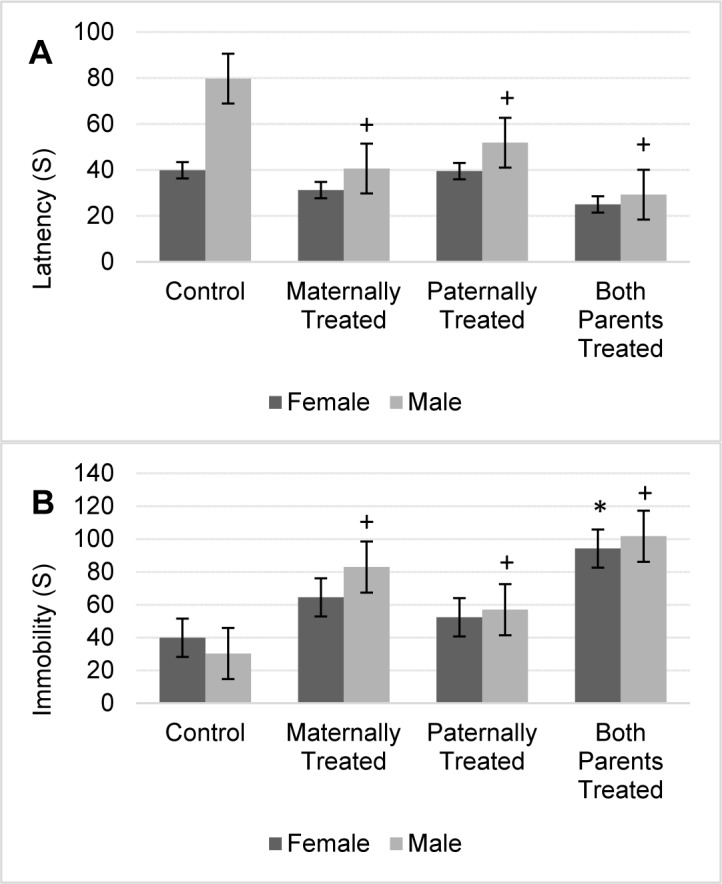
Figure 5: The mean of the total time of writhing in the animals were represented in (A), and a total number of writhing was represented in (B). Results of ANOVA test revealed, that there is a significant difference between sexes for total number of writhing in the acetic acid test (F (1, 56) = 4.25, p< 0.05). However, the analysis revealed that there is no significant difference between sexes for the total time of writhing in the acetic acid test. The average of each group was represented in offspring of the control group (Control), offspring that maternally were treated by morphine (Maternally Treated), offspring that paternally treated by morphine (Paternally Treated) and offspring that both parents were treated by morphine (Both Parents Treated). Results of ANOVA test revealed, that there is a significant difference between sexes for a total number of writhing (p< 0.05). Results of post-hoc analysis revealed that in comparison with the control group, the total time and a total number of writhing in the female offspring with (*) sign significantly was reduced (p< 0.05). Moreover, results of post-hoc analysis revealed that in comparison with the control group, the total time and a total number of writhing in the male offspring with (+) sign significantly was reduced (p< 0.05).

Results showed there is no significant difference in the total time of writhing between sexes. However the total time of writhing was significant different among the offspring (F (3, 56) = 34.53, P < 0.01). Results of mean comparison showed that in average the total time of writhing in the offspring with at least one morphine exposed parent significantly was reduced. However, locomotion in offspring with either parents or maternally exposed parents significantly was lower. Moreover, the results revealed that in male offspring with at least one morphine-treated parent, the total time of writhing significantly was reduced (Figure 5A(Fig. 5)). Results revealed that female offspring that either maternally or both parents were exposed to morphine, the total time of writhing significantly was reduced. However, there is no significant difference in the total time of writhing in female offspring that paternally were treated by morphine (Figure 5A(Fig. 5)). 

Furthermore, results showed there is a significant difference in the total number of writhing between sexes (F (1, 56) = 4.25, P < 0.05) and offspring (F (3, 56) = 42.28, P < 0.01). The results of mean comparison test showed that in average the total number of writhing in the offspring with at least one morphine exposed parent was significantly reduced, however, lowest total number of writhing was observed in the offspring that both parents were exposed to morphine (Figure 5B(Fig. 5)).

Moreover, the results showed that the interaction between offspring and sexes was significant (F (3, 56) = 5.73, P < 0.01). Results showed that in comparison to the control group, the total number of writhing in male and female offspring with at least one morphine-treated parent significantly was reduced (Figure 5B(Fig. 5)). Results showed that the lowest number of writhing was observed in male and female offspring that both parents were treated by morphine.

#### Formalin test

The results showed that there is no significant difference between sexes for acute pain perception. Furthermore, there is a significant difference among the offspring for acute pain perception (F (3, 56) = 3.52, P < 0.05). The results of mean comparison test showed that in average the acute pain in the offspring with both parents or maternally morphine exposed parents significantly was reduced. Moreover, the results of mean comparison confirmed that in the offspring that paternally exposed to morphine the average of acute pain perception, statistically was equal to average of acute pain perception in the control group. 

**Figure 6:**
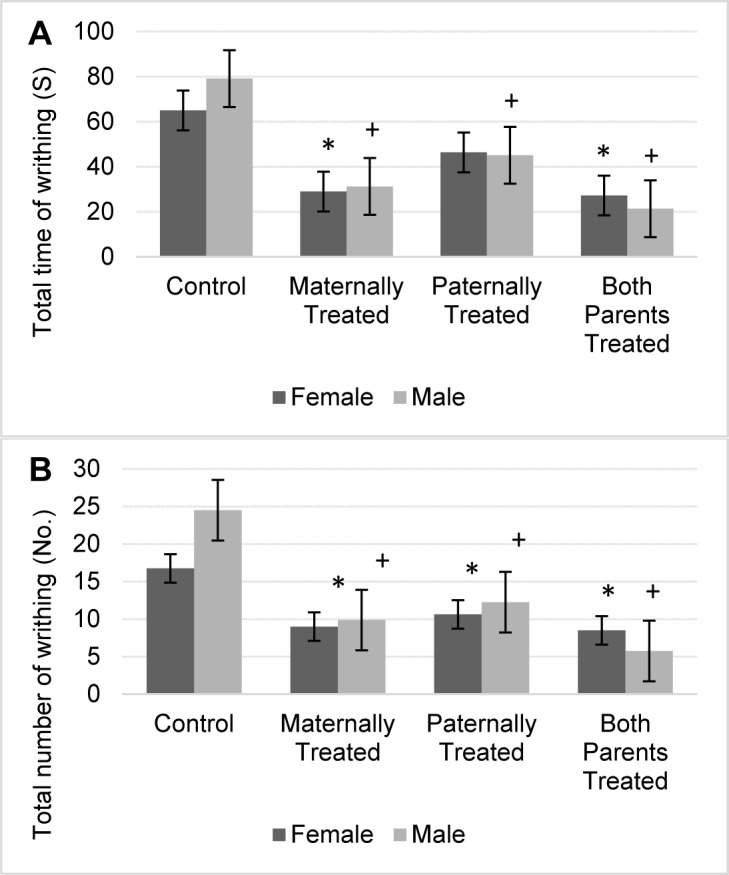
The figure shows the mean of each group for the acute pain test (A) and persistent pain test (B). Results of ANOVA test revealed that there is no significant difference between sexes for acute and persistent pain of rats in the formalin test. The average of each group was represented in offspring of the control group (Control), offspring that maternally were treated by morphine (Maternally Treated), offspring that paternally were treated by morphine (Paternally Treated) and offspring that both parents were treated by morphine (Both Parents Treated). Results of post-hoc analysis revealed that in comparison with the control group, the acute and persistent pain in the male offspring with (+) sign significantly was reduced (p< 0.05). The results of post-hoc analysis revealed that for the acute and persistent pain, there is no significant difference between the female offspring of morphine-abstinent parent(s) with the control group.

Moreover, the interaction between offspring and sexes in the acute pain perception was significantly different (F (3, 56) = 6.42, P < 0.01). The results showed that acute nociception in male offspring with at least one morphine-treated parent significantly were reduced. Moreover, there is no significant difference between female offspring with different parental morphine-exposure (Figure 6A(Fig. 6)). Moreover, results showed male offspring in control group had a higher tolerance for acute nociception in comparison with females, while the lowest acute nociception was observed in male offspring that both parents were treated by morphine (Figure 6A(Fig. 6)). 

Moreover, there is no significant difference between sexes for the perception of chronic pain. However, there is a significant difference among groups of the offspring for the perception of persistent pain (F (3, 56) = 6.00, P < 0.01). The results of mean comparison test showed that in average the perception of chronic pain in the offspring with both parents or paternally morphine exposed parents significantly was reduced. Moreover, the results of mean comparison confirmed that in the offspring that maternally exposed to morphine the average of chronic pain perception, statistically was equal to the average of chronic pain perception in the control group.

In addition, the interaction between offspring and sexes in the perception of persistent pain was significantly different (F (3, 56) = 3.21, P < 0.05). The lowest chronic pain perception was observed in male offspring that both parents were treated by morphine. The perception of chronic pain in the offspring that paternally or maternally were treated by morphine were not significantly different than the control group (Figure 6B(Fig. 6)).

For more results see the Supplementary data.

## Discussion

The results revealed that male offspring of morphine-exposed animals generally have higher behavioral changes in comparison to female offspring. The summary of all behavioral changes of the offspring is represented in Table 2(Tab. 2).

### Passive avoidance memory 

The effect of parental morphine exposure on avoidance memory of the offspring rarely has been discussed. Furthermore, the effect of epigenetic factors on memory formation is poorly understood (Tuesta and Zhang, 2014[71]). Our finding revealed that there is no significant difference between sexes and avoidance memory in male and female offspring with at least one morphine-treated parent. Moreover, the lowest value of avoidance memory was observed in male offspring that were maternally treated by morphine. The pioneer studies regarding the memory formation have been utilized contextual fear condition that produces long-lasting and robust avoidance memory in rodents. The drugs of abuse influences memory through the dopaminergic and glutamatergic pathways in the brain which makes an aberrant memory for the individual with the drugs of abuse (Torregrossa et al., 2011[69]). Evidence showed that epigenetic is an important mechanisms underlying memory storage and consolidation (Day and Sweatt, 2011[23]). Accumulative evidence suggest that paternal exposure to cocaine through epigenetic mechanisms cause changes in the memory of female offspring (He et al., 2006[32]; Day and Sweatt, 2011[23]).

The accumulative evidence suggests that parental morphine exposure could impair memory function in the offspring. The evidence suggests that parental morphine exposure exacerbates apoptosis and reduces pyramidal neurons in hippocampus (Ghafari and Golalipour, 2014[29]; Karkhah et al., 2017[39]). Another evidence revealed that morphine mediates the physiological changes in the synaptic plasticity in the hippocampus (Sarkaki et al., 2008[60]). Moreover, result shows a significant reduction in hippocampus density of the offspring that parentally exposed to morphine. Morphine exposure changes neural cell proliferation and promotes apoptosis through Mu receptors (Willner et al., 2014[74]). Moreover, morphine exposure in the prenatal lifetime could impair passive avoidance memory toghether by increasing apoptosis in the hippocampus and decreasing expression of brain derived neurotrophic factors (BDNF) (Nasiraei-Moghadam et al., 2013[50]).

Effect of parental morphine exposure on avoidance memory in different sexes rarely has been discussed. The pioneer studies suggest that parental morphine exposure has distinct effect on both sexes, however reversal of memory deficit more likely happens in the male offspring (Nasiraei-Moghadam et al., 2013[50]). The X chromosome inactivation could be another mechanism to equalize X linked gene expression in female and male (Jaenisch and Bird, 2003[36]). 

In summary, environment, genetic and synaptic plasticity are a series of changes that could affect the formation of long-term memory. Some of these changes arise during learning and they are subsequently retained in the next generation.

### Anxiety-like behavior

Our finding revealed a significant difference between sexes in latency and locomotion according to the forced swim test and open-field test. Thus, it may show that anxiety-like behaviors in the offspring of morphine-treated parents are significantly different between female and male offspring. Moreover, anxiety-like behavior was reduced significantly in male offspring with morphine-treated parent(s). However, anxiety-like behaviors such as locomotion increased significantly in female offspring that were abstinent to morphine either maternally or both of parents. Furthermore, the average of rearing was significantly reduced in female offspring that were treated by morphine paternally that might indicate a critical role of maternal inheritance for change in locomotion. On the other hand, in male offspring with at least one morphine-treated parent, the latency to immobility was reduced significantly. While in female offspring, latency to immobility showed no significant difference. 

It was reported previously that the offspring in morphine-treated parents might have neurochemical and neurophysiological disorders (Cicero et al., 1991[19]; Sarkaki et al., 2008[60]). Moreover, it was suggested that the cortisol and corticosterone level increased in the morphine-treated rats with naloxone-induced withdrawal. The increase in the level of cortisol eventually stimulated hypothalamus-pituitary axis (HPA) that may lead to the higher anxiety level (Houshyar et al., 2001[34]; Perrine et al., 2008[54]; Motaghinejad et al., 2014[48]). Evidence suggests that withdrawal symptoms are comorbid with anxiety and depression that suggest morphine dependency could induce motor deficit and anxiety-like behavior. It was concluded that morphine influences through the opioid receptors and improves anxiety-like behaviors (Georges et al., 2000[28]; Valverde et al., 2004[72]; Patti et al., 2005[53]; Zhang and Schulteis, 2008[80]). 

Differences between sexes of the offspring that parentally were exposed to morphine rarely has been discussed. The pioneer studies revealed that males exhibit greater symptoms of withdrawal compared to females (Becker et al., 2017[11]). Moreover, males have a longer period of abstinence, and females are more likely to relapse compared to males (Becker et al., 2017[11]). Furthermore, ovarian hormones influence the drug taking in female rats. It was reported that rats worked harder to receive cocaine during the estrus phase (Roberts et al., 1989[57]; Becker and Hu, 2008[9]). The alteration of the hormone level during the estrous cycle might enhance the initial reinforcement effect that the lad females get from drugs abuse. However, once the addictive behavior was established, the hormone does not play a role (Becker et al., 2017[11]). Moreover, females exhibit higher reinstatement in drugs and morphine seeking compared to the males (Anker and Carroll, 2010[6]; Feltenstein et al., 2011[25]; Becker and Koob, 2016[10]). Furthermore, it was reported that parental opioid exposure has led to the anxiety-like behavior in the offspring (Klausz et al., 2011[43]).

In male rats, morphine exposure has delayed sexual maturation and has led to the smaller size of offspring and it has induced a significant endocrine change in the offspring (Cicero et al., 1991[19]). Another evidence showed that the varieties of psychopathologies such as anxiety, substance abuse, and suicidal attacks are common among children with addicted parents (Balsa et al., 2009[8]). The evidence showed that parental morphine exposure decreases long-term anxiety in the hippocampus (Yang et al., 2003[75]). These changes in the neuronal plasticity in the hippocampus also may influence anxiety-like behavior in the offspring of morphine-treated parent(s) (Miranda-Paiva et al., 2001[46]; Slamberová et al., 2001[66]). Our previous study showed that parental morphine exposure before gestation changes the behavior of the offspring (Torkaman-Boutorabi et al., 2019[68]). The evidence revealed that mu, delta, and kappa opioid receptors have strong control over the behavioral process. Especially the delta and kappa opioid receptors have a distinct antidepressant potential (Lutz and Kieffer, 2013[44]). Mu, kappa, and delta opioid receptors are expressed in the oocyte (Agirregoitia et al., 2012[1]). The mu opioid receptor and beta-endorphin are expressed in the male reproductive organ (Albrizio et al., 2006[4]). Furthermore, gonadal hormones such as estrogen in female rats enhanced the effect of drugs whereas progesterone showed the opposite effect (Becker and Hu, 2008[9]; Quinones-Jenab and Jenab, 2010[56]; Anker and Carroll, 2011[5]). 

Regarding this background, morphine exposure even before gestation may influence transgenerational inheritance causing the anxiety-like behaviors. The intergenerational effect of morphine on gene transcription and hormonal influence is contributed to the behavioral difference in female and male rats. Consequently, our results suggest that morphine could induce the transgenerational effect and it alters the endogenous opioid system in the offspring. 

### Pain experience 

Our finding showed that visceral, acute and persistent nociception was decreased, especially in male offspring with at least one morphine-treated parent. Our results revealed that the differences between sexes were significant only in the formalin test of persistent pain and the total number of writhes in writhing test. The lowest number of writhes was observed in the offspring where both parents were morphine-treated. While the lowest perception of acute and chronic pain was observed in male offspring where both parents were morphine-treated. 

The pioneer studies show that exposure to drugs is different in males and female. For instance, males take the drug as an engagement in risky behaviors in order to be a part of the group, while females tend to experience the pleasurable response of the drugs (Becker et al., 2017[11]). Evidence showed that males and females are different in nociception; the females had higher pain perception and they showed a more negative response to the pain (Fillingim and Gear 2004[27]). The difference is generally accepted although the mechanism of this dimorphism is not clear. Moreover, female rats intensively react to a stressor compared to males due to the change in the glucocorticoid negative feedback inhibition (Steingart et al., 1998[67]). It is noteworthy that the effects of opioids on hormonal excretion have been rarely measured (Seyfried and Hester, 2012[64]) and the peripheral nociceptive neurons are sensitive to opiates (Collier et al., 1968[20]). The evidence suggests that drug exposure induces reward circuit adaptation in the brain regions (Schmidt et al., 2003[62]). Moreover, pain perception would cause sustained activation of kappa opioidergic system in the NAC that suppresses morphine which induces a rewarding effect (Narita et al., 2005[49]). The increase in the dopamine level causes decrease in the perception of the pain in the offspring. The nociception also might be related to the intensity of the pain stimulus which is characterized by the environmental condition, epigenetic and genetic factors (Elmer et al., 1998[24]; Ashabi et al., 2018[7]). In this background the reduction of pain perception in the offspring could be related to upregulation of dopamine receptor which transgenerationally inherited through epigenetic mechanisms.

Moreover, in the sperms of male subjects with opioid addiction, the expression of opioid receptor genes was changed through the epigenetic mechanisms that could inherit to the next-generation (Chorbov et al., 2011[18]). The chronic opioid misuse alters opioid receptor genes that may impair germ cell development and roles epigenetic transmission in addicted phenotypes (Chorbov et al., 2011[18]; Chidambaran et al., 2017[17]). We conclude that sex difference in the perception of pain in the offspring might be influenced by hormonal excretion, which was altered in female offspring of the morphine-treated parent. 

## Summary and Conclusion

Results suggest that there are behavioral changes based on the sex which is under transgenerational influence. Both oocyte and sperm possess opioid receptors; suggesting that the alterations in the opioid receptor's expression could transfer to the next-generation and also it suggests that the offspring of addicted parents are subjected to a greater vulnerability to psychiatric disorders (Miller et al., 2001[45]; Agirregoitia et al., 2012[1]). Moreover, some of the genes contained in the Y and X chromosome may produce different protein isoforms. Furthermore, XX cells are subjected to maternal imprint while XY cells may be influenced by maternal and paternal imprint (Bourc'his and Proudhon, 2008[14]). The major epigenetic mechanisms involved in drug addiction are DNA methylation, histone modification and microRNAs (Robison and Nestler, 2011[58]; Kanherkar et al., 2014[38]). Furthermore, cytosine methylation is required for X chromosomal inactivation, genetic impairment and normal development, which suggests the mechanism underlying different responses in male and female animals (Newell-Price et al., 2000[51]; Bird, 2008[13]). The scientific basis for medical decisions is based on the data collected from the male animals. In this study we included female rats which may increase the chance of the personalized medicine for females. In conclusion, the study revealed that the endogenous opioid system might be altered in the offspring of the morphine-treated parent(s), and epigenetic inheritance might have an important role. However, the analysis of variance signified the important role of maternal inheritance.

## Acknowledgement

This work was supported by the National Elite Foundation and Tehran University of Medical Science under Grant 94-01-159-28023 and 94-01-159-28023.

## Supplementary Material

Supplementary data

## Figures and Tables

**Table 1:**
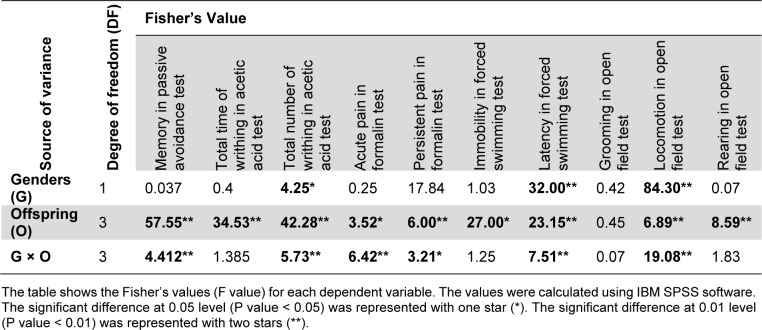
Results of two way ANOVA analysis

**Table 2:**
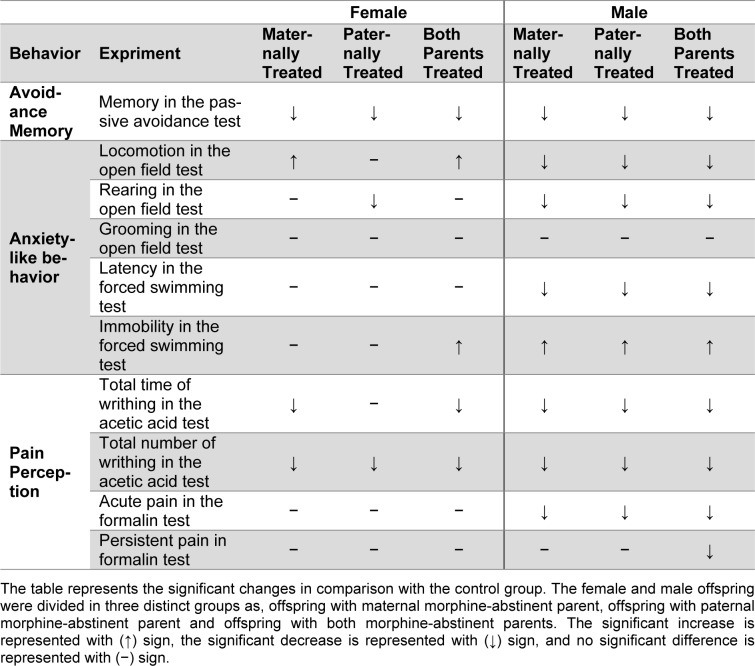
Summary of the results
