# Supplementary material for: Transgenerational influence of parental morphine exposure on pain perception, anxiety-like behavior and passive avoidance memory among male and female offspring of Wistar rats
Source: EXCLI J. 2019 Nov 5;18:1019–36. doi: 10.17179/excli2019-1845 (PMC6868917; doi:10.17179/excli2019-1845)
Supplement: Supplementary data [file EXCLI-18-1019-s-001.pdf]

EXCLI Journal 2019;18:1019-1036 – ISSN 1611-2156
Received: September 26, 2019, accepted: October 29, 2019, published: November 05, 2019

Supplementary data to:

TRANSGENERATIONAL INFLUENCE OF PARENTAL MORPHINE EXPOSURE ON PAIN PERCEPTION, ANXIETY-LIKE BEHAVIOR AND PASSIVE AVOIDANCE MEMORY AMONG MALE AND FEMALE OFFSPRING OF WISTAR RATS

Hamid Ahmadian-Moghadam[1], Mitra-Sadat Sadat-Shirazi[1], Fereshteh Seifi[2], Saba Niknamfar[2], Ardeshir Akbarabadi[1,3], Heidar Toolee[4], Mohammad-Reza Zarrindast[1,5,6,*]

[1] Iranian National Center for Addiction Studies, Tehran University of Medical Sciences, Tehran, Iran
[2] Biology Department, Faculty of Biological Sciences, Islamic Azad University, North Tehran Branch, Tehran, Iran
[3] Department of Veterinary Medicine, Garmsar Branch, Islamic Azad University, Garmsar, Iran
[4] Department of Anatomy, School of Medicine, Tehran University of Medical Sciences, Tehran, Iran
[5] Department of Pharmacology, School of Medicine, Tehran University of Medical Sciences, Tehran, Iran
[6] Endocrinology and Metabolism Research Institute, Tehran University of Medical Science, Tehran, Iran

* Corresponding author: M.R. Zarrindast, Department of Pharmacology, School of Medicine, Tehran University of Medical Sciences, Tehran, Iran, P.O.Box: 13145-784, Tel: +9821-66402569, Fax: +9821-66402569, E-mail: zarinmr@ams.ac.ir

http://dx.doi.org/10.17179/excli2019-1845

This is an Open Access article distributed under the terms of the Creative Commons Attribution License (http://creativecommons.org/licenses/by/4.0/).

Received: September 26, 2019, accepted: October 29, 2019, published: November 05, 2019

ABSTRACT

The main objective of the current study is to evaluate the effect of parental morphine exposure on avoidance memory, morphine preference and depressive-like behavior of offspring. The total of 32 males and 32 females were used for mating. The animals were treated with morphine. The offspring according to their parental morphine treatment was divided into four groups (n=16) including paternally treated, maternally treated, both of parents treated and naïve animals. The pain perception, depressive-like behavior, and avoidance memory were evaluated in the offspring. In the current study, the total of 256 offspring was used for the experiments (4 tasks × 4 groups of offspring × 8 female offspring × 8 male offspring). The chance of false positive results was reduced by normality analysis using Kolmogorov-Smirnov (K-S) test. The normal data with K-S value more than 0.01, were subjected to two-way ANOVA analysis followed by Dunnett's post-hoc mean comparison test. The p-values lower than 0.05 were considered significant. All statistical analyses were conducted using IBM SPSS 21 software.

Keywords: Addiction, epigenetic, pain, depression, memory, morphine

Specifications Table

Subject	Neuroscience: behavior
Specific subject area	Depressive live behavior, Pain perception, Memory, Epigenetics
Type of data	Tables
How data were acquired	Avoidance memory: Passive-avoidance memory test<br>Depression like behavior: Open-Field Test and Forced Swimming Test<br>Pain perception: Writhing test and Formalin test
Data format	Raw<br>Analyzed
Parameters for data collection	Gender (female and male)<br>Memory retention in avoidance memory test<br>Total number of movement in open field test<br>Total number of rearing in open field test<br>Total number of grooming in open field test<br>Total time of immobility in forced swimming test<br>Time of latency to immobility in forced swimming test<br>Total number of licking in 20 min in formalin test<br>Total number of licking in 10 min in formalin test<br>Total number of writhing in acetic acid test<br>Total time of writhnig in acetic acid test.
Description of data collection	The total of 32 males and 32 females were used for mating. The animals were treated with morphine. The offspring according to their parental morphine treatment was divided into four groups (n=16) including paternally treated, maternally treated, both of parents treated and naïve animals. The pain perception, depressive-like behavior, and avoidance memory were evaluated in the offspring. In the current study, the total of 256 offspring was used for the experiments (4 tasks × 4 groups of offspring × 8 female offspring × 8 male offspring).
Data source location	Iranian National Center for Addiction Studies, Tehran University of Medical Sciences, Tehran, Iran
Data accessibility	The data are hosted 'With the article'
Related research article	Akbarabadi A, Niknamfar S, Vousooghi N, Sadat-Shirazi M-SS, Toolee H, Zarrindast M-RR. Effect of rat parental morphine exposure on passive avoidance memory and morphine conditioned place preference in male offspring. Physiol Behav 2018;184:143–9. doi:10.1016/j.physbeh.2017.11.024.

EXCLI Journal 2019;18:1019-1036 – ISSN 1611-2156
Received: September 26, 2019, accepted: October 29, 2019, published: November 05, 2019

Value of the data

- The data would be useful to assess effect of parental morphine exposure on the behavior of the offspring
- The comparison between female and male offspring that parentally were exposed to addictive drugs rarely has been discussed.
- The data would be useful for the researchers interested in epigenetic or transgenerational influence of parental morphine exposure on the child
- The data could be useful for researchers who are interested on the long-term effect of opioids exposure and other addictive substances on the individuals.

EXPERIMENTAL DESIGN, MATERIALS, AND METHODS

Animals

All experimental procedures were in agreement with the regulations of the experimental animal ethics at Tehran University of Medical Sciences ethics committee. Wistar albino rats, weighing between 200 to 220 gram were purchased from Pasture Institute, Tehran, Iran. The rats were exposed to morphine according to a modified protocol of Akbarabadi and colleagues (Akbarabadi et al., 2018). The total of 32 males and 32 females were used for mating and morphine treatment. The total of 128 male and 128 female offspring were used for the experiments (4 tasks × 4 groups of offspring × 8 female × 8 male). The animals were maintained in Plexiglas cages (n=4) with free access to fresh water and food at constant temperature 22±2 ºC and light/dark cycle (07:00–19:00 h). The biological father was removed before the birth of the offspring and the biological mother was kept until the end of the breastfeeding period. The experiments were started when the offspring were reached to 8 weeks old. Moreover, female rats were tested on the diestrous phase of the estrous cycle and vaginal smear test was monitored daily. Moreover, to avoid potential confounding factory the offspring were no over-represented in the experiments. Furthermore one week before starting of the experiment the animals were picked up daily to reduce handling anxiety (Gouveia and Hurst, 2013).

Drugs

In this study morphine sulfate (Temad Co., Tehran, Iran), naloxone hydrochloride (Sigma-Aldrich), and sucrose (Merck) were used.

Parental morphine exposure

Twenty-four male and twenty-four female Wistar rats were exposed to the treatment of oral morphine sulfate according to the protocol described earlier (Akbarabadi et al., 2018). Morphine was given in the drinking fluid in the range of 0.1 to 0.4 $mg.ml^{-1}$ in 48 intervals for up to three weeks (Figure 1A). Sucrose (2 %) was added to diminish the bitter taste of morphine. Eight male and eight female rats were considered as the control group which only received sucrose (2 %). Naloxone was administrated intraperitoneally (IP) for all of the animals to confirm the morphine dependence. Withdrawal symptoms and the average of morphine consumption were recorded (Figure 1B and C).

EXCLI Journal 2019;18:1019-1036 – ISSN 1611-2156
Received: September 26, 2019, accepted: October 29, 2019, published: November 05, 2019

Mating protocol

Ten days after the last morphine administration, the animals were assigned for mating. The offspring of the animals was arranged in four groups as offspring of healthy parents (naive), offspring of morphine-treated female and healthy male rats (maternally treated), offspring of morphine-treated male and healthy female rats (paternally treated) and offspring of morphine-treated male and female rats (both of parents were treated).

Avoidance memory

Passive-avoidance memory test

The passive avoidance memory is evaluated according to the protocol that is described earlier (Akbarabadi et al., 2018). The avoidance memory apparatus consists of a box with two compartments that were separated by a guillotine door. In the learning trial, the animals were placed in the light compartment and were allowed to cross the dark compartment, the guillotine door was closed and received an electric shock via gird floor (50 Hz, 1 mA, and 5s). The latency time of each animal to cross to the dark compartment was recorded. The experiment was repeated and in case of no entrance to the dark compartment within 120s, a successful acquisition of avoidance memory was recorded. The animals with successful acquisition were subjected to the test trial. In the test trial, each animal was placed in the light compartment and the door was opened. The step-through latency for crossing to the dark compartment was recorded for each animal. The testing trial ended either when the animal entered into the dark compartment or remained in the light compartment until the cut-off time (300 s).

Depressive-like behavior

Open-field test

The open-field test is a behavioral test to evaluate depressive-like behavior based on the locomotor activity (Damián et al., 2014; Motaghinejad et al., 2016). The open-field apparatus consists of a plexiglass square box with walls to reduce outside noise and light. Each rat was placed at the center of the apparatus and left to move freely for 10 min. The number of times that the animal preened its fur or tail with its mouth or forepaws (grooming), square crossed (locomotion) and the number of times that a rat reared up on its hind limbs (rearing) were recorded. After each monitor, the cage was cleaned with 70 % ethanol solution and left to dry.

Forced swimming test

The rats that were used for the open-field test were re-used in the forced swimming test. Forced swimming test is conducted to assess depressive-like behavior acording to the protocol described earlier (Porsolt et al., 1977). Briefly, the rat was placed on a Plexiglas cylinder (60 × 30 cm) that was filled with water (25 °C) to the height of 30 cm. In trial day the rats were habituated to the environment by swimming in the cylinder for 5 min in 24 h before test day. In the test day each rat was allowed to swim for 5 min while a video camera recorded from above. The latency to immobility and the total time of immobility were measured as an index of depressive-like behavior.

Pain perception

Writhing test

The writhing test is a method to evaluate nociception. Each animal was placed on a small observation chamber. After 10 min of habituation a volume of 10 $ml.kg^{-1}$ acetic acid (0.8 %) was i.p administrated. The nociceptive behavior characterized by abdominal contraction known

as writhing, is described as an exaggerated extension into the abdomen combined with the out-stretching of hind limbs. Five minutes following the administration, the number of writhing and total time of writhing was recorded over 10 minutes (Singh et al., 1983).

Formalin test

The formalin test was performed to evaluate acute and chronic pain. The pain is induced by applying 0.1 ml of 2.5 % formalin (Merck, Germany) into the dorsal surface of the left hind paw of each rat, and the rats were placed in an observation chamber with a mirror mounted on three sides to allow a clear view of the paws. The total times that each rat spent licking the injected paws were recorded. Acute pain as a result of nociceptor stimulation was observed in 1–10 min interval and the persistent pain was observed in 20–40 min interval of formalin injection (Hunskaar et al., 1985).

Statistical analysis

The chance of false positive results was reduced by normality analysis using Kolmogorov-Smirnov (K-S) test. The normal data with K-S value more than 0.01, were subjected to two-way ANOVA analysis followed by Dunnett's post-hoc mean comparison test. The p-values lower than 0.05 were considered significant. All statistical analyses were conducted using IBM SPSS 21 software. The output of the statistical analysis is summarized in Table 2.

EXCLI Journal 2019;18:1019-1036 – ISSN 1611-2156
Received: September 26, 2019, accepted: October 29, 2019, published: November 05, 2019

Data:

Table 1: Raw data of the experiment. A: list of groups, (CTL: Control, PTN: paternally treated offspring, maternally treated offspring, BTH: both parents treated with morphine), G: Gender (F: female, M: Male), MEM: memory in avoidance memory test, LOC: total number of movement in open field test, REA: total number of rearing in open field test, GRO: total number of grooming in open field test, FST: total time of immobility in forced swimming test, FSI: time of latency to immobility in forced swimming test, FCH: total number of licking in 20 min in formalin test, FAC: total number of licking in 10 min in formalin test, ATN: Total number of writhing in acetic acid test, ATT: total time of writhing in acetic acid test.

A	G	MEM	DEPRESSION					PAIN			
			LOC	REA	GRO	FST	FSI	FCH	FAC	ATN	ATT
CTL	F	300	23	16	5	35	44	305	58	17	61
CTL	F	300	21	16	4	34	42	221	53	15	66
CTL	F	121	10	3	10	55	25	220	95	15	64
CTL	F	241	2	5	2	45	38	227	45	14	67
CTL	F	90	22	8	3	22	60	237	27	30	105
CTL	F	300	11	10	3	33	60	135	28	16	68
CTL	F	300	10	4	5	65	20	247	23	11	24
CTL	F	236	19	9	8	30	45	227	47	16	65
CTL	M	244	21	13	3	90	15	474	77	28	115
CTL	M	267	28	12	5	75	43	310	100	25	75
CTL	M	300	16	7	8	87	14	356	75	16	47
CTL	M	300	18	15	4	85	21	654	94	21	68
CTL	M	300	40	16	5	62	42	280	95	30	88
CTL	M	300	11	7	8	92	30	363	72	29	85
CTL	M	258	22	17	1	82	24	275	90	23	79
CTL	M	258	22	12	7	65	53	387	87	24	76
PTN	F	300	22	1	2	25	65	282	45	8	36
PTN	F	50	33	11	3	45	54	259	91	3	44
PTN	F	22	10	0	10	55	30	135	41	12	56
PTN	F	16	17	0	3	61	28	127	37	15	72
PTN	F	45	29	2	8	40	62	392	54	14	48
PTN	F	43	50	2	8	50	32	190	82	8	23
PTN	F	110	38	2	7	20	72	211	49	13	46
PTN	F	300	31	2	4	20	76	251	34	12	46
PTN	M	2	15	3	0	45	85	325	42	10	27
PTN	M	49	7	12	5	65	36	205	55	19	63
PTN	M	5	0	5	9	53	55	377	86	13	43
PTN	M	32	0	1	6	60	30	272	26	11	42
PTN	M	29	4	6	10	50	60	384	73	9	27
PTN	M	2	6	2	8	48	70	365	68	10	26
PTN	M	21	9	0	15	54	50	155	17	14	88
PTN	M	55	5	4	2	40	70	340	52	12	45
MTN	F	1	21	3	5	25	55	300	70	8	29

EXCLI Journal 2019;18:1019-1036 – ISSN 1611-2156
Received: September 26, 2019, accepted: October 29, 2019, published: November 05, 2019

Table 1 (cont.)

A	G	MEM	DEPRESSION				PAIN			
			LOC	REA	GRO	FST	FSI	FCH	FAC	ATN	ATT
MTN	F	5	30	7	6	20	82	294	62	7	27
MTN	F	40	70	9	4	27	62	74	21	8	30
MTN	F	91	47	9	6	21	93	85	32	15	29
MTN	F	10	48	10	14	45	44	174	42	7	22
MTN	F	33	48	4	3	30	67	102	72	9	37
MTN	F	15	31	6	6	55	38	54	14	9	29
MTN	F	32	45	13	4	27	75	154	44	9	29
MTN	M	19	5	0	0	35	80	277	66	16	51
MTN	M	32	14	6	9	35	105	470	90	7	21
MTN	M	12	2	1	4	30	110	230	16	6	19
MTN	M	42	20	12	4	35	98	123	65	11	35
MTN	M	31	24	11	7	40	77	265	12	9	28
MTN	M	11	2	1	5	60	57	243	54	10	35
MTN	M	15	11	4	16	50	57	490	18	9	25
MTN	M	38	11	5	7	40	80	290	45	11	36
BTH	F	2	52	10	3	20	82	244	50	10	39
BTH	F	5	37	8	5	50	35	177	63	7	19
BTH	F	30	40	0	3	20	113	185	73	9	18
BTH	F	55	45	0	8	22	107	127	25	8	23
BTH	F	21	64	12	5	18	110	203	107	10	34
BTH	F	1	55	3	8	15	120	255	81	8	31
BTH	F	47	56	15	8	25	97	137	23	8	27
BTH	F	30	49	6	8	30	90	182	47	8	27
BTH	M	61	0	1	2	40	71	190	32	6	18
BTH	M	98	13	5	3	30	105	184	24	1	19
BTH	M	61	12	6	8	25	113	201	25	1	17
BTH	M	10	17	5	12	28	100	124	44	8	24
BTH	M	47	10	11	6	32	98	201	35	7	24
BTH	M	11	6	6	8	38	80	187	26	9	27
BTH	M	115	12	4	3	15	165	135	31	7	23
BTH	M	80	10	5	9	26	82	209	31	7	19

EXCLI Journal 2019;18:1019-1036 – ISSN 1611-2156
Received: September 26, 2019, accepted: October 29, 2019, published: November 05, 2019

Table 2: Output of software for two way ANOVA analysis

Source of variance	Degree of freedom (DF)	Fisher's Value									
		Memory in passive avoidance test	Total time of writhing in acetic acid test	Total number of writhing in acetic acid test	Acute pain in formalin test	Persistent pain in formalin test	Immobility in forced swimming test	Latency in forced swimming test	Grooming in open field test	Locomotion in open field test	Rearing in open field test
**Genders (G)**	1	0.037	0.4	**4.25***	0.25	17.84	1.03	**32.00****	0.42	**84.30****	0.07
**Offspring (O)**	3	**57.55****	**34.53****	**42.28****	**3.52***	**6.00****	**27.00***	**23.15****	0.45	**6.89****	**8.59****
**G × O**	3	**4.412****	1.385	**5.73****	**6.42****	**3.21***	1.25	**7.51****	0.07	**19.08****	1.83

The table shows the Fisher's values (F value) for each dependent variable. The values were calculated using IBM SPSS software. The significant difference at 0.05 level (P value < 0.05) was represented with one star (*). The significant difference at 0.01 level (P value < 0.01) was represented with two stars (**).

Acknowledgments

This work was supported by the National Elite Foundation and Tehran University of Medical Science under Grant 94-01-159-28023 and 94-01-159-28023.

REFERENCES

Akbarabadi A, Niknamfar S, Vousooghi N, Sadat-Shirazi M-SS, Toolee H, Zarrindast M-RR. Effect of rat parental morphine exposure on passive avoidance memory and morphine conditioned place preference in male offspring. Physiol Behav. 2018;184:143–9. http://linkinghub.elsevier.com/retrieve/pii/S0031938417304158

Damián JP, Acosta V, Da Cuña M, Ramírez I, Oddone N, Zambrana A, et al. Effect of resveratrol on behavioral performance of streptozotocin-induced diabetic mice in anxiety tests. Exp Anim. 2014;63:277–87. http://www.ncbi.nlm.nih.gov/pubmed/25077757

Gouveia K, Hurst JL. Reducing mouse anxiety during handling: effect of experience with handling tunnels. PLoS One. 2013;8:e66401. http://dx.plos.org/10.1371/journal.pone.0066401. accessed 7 August 2017

Hunskaar S, Fasmer OB, Hole K. Formalin test in mice, a useful technique for evaluating mild analgesics. J Neurosci Methods. 1985;14:69–76. http://www.ncbi.nlm.nih.gov/pubmed/4033190

Motaghinejad M, Fatima S, Banifazl S, Bangash MY, Karimian M. Study of the effects of controlled morphine administration for treatment of anxiety, depression and cognition impairment in morphine-addicted rats. Adv Biomed Res. 2016;5:178. http://www.ncbi.nlm.nih.gov/pubmed/28028518

Porsolt RD, Le Pichon M, Jalfre M. Depression: a new animal model sensitive to antidepressant treatments. Nature. 1977;266:730–2. http://www.ncbi.nlm.nih.gov/pubmed/559941

Singh PP, Junnarkar AY, Rao CS, Varma RK, Shridhar DR. Acetic acid and phenylquinone writhing test: a critical study in mice. Methods Find Exp Clin Pharmacol. 1983;5:601–6. http://www.ncbi.nlm.nih.gov/pubmed/6668969
